# Identifying the role of (dis)inhibition in the vicious cycle of substance use through ecological momentary assessment and resting-state fMRI

**DOI:** 10.1038/s41398-024-02949-1

**Published:** 2024-06-19

**Authors:** Valentine Chirokoff, Sylvie Berthoz, Melina Fatseas, David Misdrahi, Maud Dupuy, Majd Abdallah, Fuschia Serre, Marc Auriacombe, Adolf Pfefferbaum, Edith V. Sullivan, Sandra Chanraud

**Affiliations:** 1 Univ. Bordeaux, INCIA CNRS-UMR 5287, Bordeaux, France; 2 EPHE, PSL Research University, Paris, France; 3 Institut Mutualiste Montsouris, Department of Psychiatry for Adolescents and Young Adults, Paris, France; 4 CH Charles Perrens, Bordeaux, France; 5 CHU Bordeaux, Bordeaux, France; 6 Bordeaux University, CNRS, Bordeaux Bioinformatics Center, IBGC UMR 5095, Bordeaux, France; 7 Groupe d’Imagerie Neurofonctionnelle, Institut des Maladies Neurodégénératives-UMR 5293, CNRS, University of Bordeaux, Bordeaux, France; 8 University of Bordeaux, CNRS UMR 6033– Sleep, Addiction and Neuropsychiatry (SANPSY), Bordeaux, France; 9 Center for Health Sciences, SRI International, Menlo Park, CA USA; 10 Department of Psychiatry & Behavioral Sciences, Stanford University School of Medicine, Stanford, CA USA

**Keywords:** Addiction, Predictive markers, Human behaviour

## Abstract

Functional inhibition is known to improve treatment outcomes in substance use disorder (SUD), potentially through craving management enabled by underlying cerebral integrity. Whereas treatment is challenged by a multitude of substances that patients often use, no study has yet unraveled if inhibition and related cerebral integrity could prevent relapse from multiples substances, that is, one’s primary drug of choice and secondary ones. Individuals with primary alcohol, cannabis, or tobacco use disorders completed intensive Ecological Momentary Assessment (EMA) coupled with resting-state functional MRI (rs-fMRI) to characterize the extent to which inhibition and cerebral substrates interact with craving and use of primary and any substances. Participants were 64 patients with SUD and 35 healthy controls who completed one week EMA using Smartphones to report 5 times daily their craving intensity and substance use and to complete Stroop inhibition testing twice daily. Subsamples of 40 patients with SUD and 34 control individuals underwent rs-fMRI. Mixed Model Analysis revealed that reported use of any substance by SUD individuals predicted later use of any and primary substance, whereas use of the primary substance only predicted higher use of that same substances. Craving and inhibition level independently predicted later use but did not significantly interact. Preserved inhibition performance additionally influenced use indirectly by mediating the link between subsequent uses and by being linked to rs-fMRI connectivity strength in fronto-frontal and cerebello-occipital connections. As hypothesized, preserved inhibition performance, reinforced by the integrity of inhibitory neurofunctional substrates, may partake in breaking an unhealthy substance use pattern for a primary substance but may not generalize to non-target substances or to craving management.

## Introduction

Substance use disorder (SUD) is the most prevalent form of mental illness [1, 2] and one of the major contributors to the Global Burden of Disease [3]. In this context, optimizing treatment for SUD is one of the highest priorities for translating research to benefit the individual, family, and community at large. Traditionally, SUD treatments focus directly on preventing use, in terms of quantity or frequency, to encourage patients to avoid the substance they are treated for. In recent years, a shift has been emerging towards a widening of addiction treatment objectives, to obtain a global improvement in patient functioning [4]. Among the known factors influencing such outcomes, functional inhibition appears predominant to ensure optimal treatment benefit efficiency [5, 6]. Inhibition is defined as the ability to deliberately inhibit dominant, automatic, or prepotent responses when necessary [7]. Deficits in this function have been hypothesized to be directly linked to enhanced use of the primary substance [8], through underlying cerebral alterations [9] or by invoking moderating effects of other risk factors such as craving [10]. However, most patients treated for SUD do not exhibit a single, substance use pattern and co-occuring use of other substances contributes to the morbidity and mortality of hospitalized patients [11]. This consideration highlights the importance of investigating if and how prognostic factors could protect from the use of any substance in general. As substance use [12], craving [13], and inhibition [14] fluctuate rapidly in time, the conjoint testing of their interaction requires advanced methodologies that merge neuroimaging and intensive real-time, longitudinal assessment of risk factors and executive functioning, not previously addressed.

Inhibition deficits have been depicted as a core component of SUD [15], both as consequences [16] and predispositions [17] of use. Substance misuse can produce cerebral disturbance in executive-related areas, typically frontal systems that could in turn produce an inhibition deficit, thereby leading to the vicious circle of addiction [18–20]. The imbalance caused by such deficits would lead to loss of control over use and the dysregulation craving [15, 21]. Conversely, substance abstinence can afford opportunities to enhance inhibitory control [22] and ultimate recovery of compromised neural substrates [23].

The challenge remains regarding how to identify the conditions under which inhibition is associated with treatment failure or success [24]. Use of a drug, legal or not, is known to increase the probability of use of other substances, in all ages and drug-using populations [25]. Indeed polysubstance use drastically interferes with the clinical progress potentially by precipitating relapse in the primary substance or acting as a substitution for the primary drug [26]. Such dynamic influence of use over later use along with their predictors can be assessed in real life settings with a smartphone using Ecological Momentary Assessment (EMA). The intensively repeated nature of such data collection allows investigation of time-lagged predictions of subsance use (i.e., “what comes first?”) and the complex interaction between factors of relapse, notably craving and inhibition [27]. Herein, we aimed to exploit the benefits of EMA coupled with neuroimaging data to uncover the potential of inhibitory control to avert misuse of any substance.

The aim of this EMA study was to investigate direct and indirect effects of inhibition on the use of primary substance and any substance use in patients treated for primary Alcohol, Tobacco and Cannabis Use Disorders. To test for specificity of brain-behavior relations in patients with SUD, we included a sample of healthy subjects to compare executive and rs-fMRI integrity between the groups.

## Method

### Participants

Participants were volunteers and provided their written informed consent. Financial compensation was provided with a maximum of €100 in store vouchers for the completion of both the EMA and MRI study phases. Participants with SUD were recruited at *Centre Hospitalier Charles Perrens* where they were beginning regular outpatient treatment for addiction. Hence the entirety of the experimental protocol was conducted after treatment initiation. All patients met DSM-5 criteria for a current primary alcohol, tobacco, or cannabis use disorder and could receive care including pharmacotherapy, individual behavioral treatments (relapse prevention and psychosocial support), or both. Patients exhibiting multiple SUD could be included in the study if one substance (Alcohol, Tobacco or Cannabis) was prioritized during treatment as defined by the patient and the psychiatrist. Substance-related data were assessed using a validated French version of the Addiction Severity Index [28, 29], modified to take into account tobacco addiction [30]. The Interviewer Severity Ratings from the drug (in this study, cannabis), alcohol and tobacco sections of the ASI were used to assess the severity of the addiction in the primary substance. Patients with a history or current bipolar or schizophrenia disorder (Mini International Neuropsychiatric Interview 5.0.0 (MINI [31]) were excluded; patients who presented a current comorbid depressive disorder were included. All participants had to be free from conditions incompatible with the use of a smartphone and MRI scanning.

This study was conducted in compliance with ethical standards depicted in the Helsinki declaration and approved by the ethical committee “Comité de Protection des Personnes de Sud-Ouest et Outre-Mer III» (N° 2014-A01668-39). Sample size for the MRI analyses was estimated using the package pwr available on R [32]. For a linear regression with 2 predictors (inhibition performance and age) with a minimal power of 0.80, an alpha level of 0.05 to detect a medium effect size (R^2^ = 0.20), we estimated that 41 subject would be needed.

### Procedure

The feasibility and validity of the EMA methodology, including mobile cognitive testing (adaptation of Stroop test) were previously demonstrated in patients with SUD [33]. All participants were trained to operate the smartphone (Samsung Galaxy S with a 10.6 cm screen, 12-point font size) assigned for the study before the 7 consecutive days of EMA assessments. They were instructed to carry the smartphone and to respond to five electronic surveys per day randomly occurring in time intervals based on their personalized time of “beginning” and “end” of the day (resulting in approximately 3 hours between each survey). EMA assessments included questionnaires to rate craving intensity on a scale from 1 to 7 and to record use of the primary substance and any substance 5 times per day; in addition, inhibition testing occurred twice per day with the timed incongruent condition of the Stroop task. Participants had to name the ink color of 16 incongruent color names (e.g. blue written in red) as quickly as possible. Verbal responses were recorded via the EMA device in order to estimate the precise completion time in ms, with longer times indicating worse performances.

**Fig. 1:**
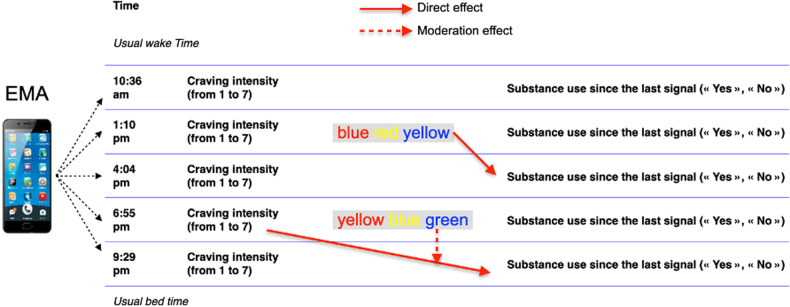
Typical day of EMA assessments and illustration of potential direct vs indirect effect of inhibition on use. Usual “wake” and “sleep” times were selected by each participant to ensure that they would be able to answer each signal. Signals occurred randomly within 5-time intervals periods and could concern craving and substance use only or could be followed by a Stroop test. Craving was assessed on a scale from 1 to 7, and Substance use was coded in a dichotomous manner (“Yes” or “No”) for the primary substance and for any substance. Stroop refers to the timed ink color naming of an incongruent color-related word (i.e., blue written in yellow ink), with longer time completion indicating a poorer inhibition performance. All variables can be used to predict the same-time assessment level or next-time assessment level (i.e., lagged in time) of another measure. This prediction can test direct effect as indicated by the continuous arrow or indirect effect, such as moderation, as indicated by the dotted arrow.

A typical day of EMA is presented in Fig. 1. A subsample of 40 patients with SUD and 34 control participants underwent neuroimaging to acquire anatomical and rs-fMRI data within the 48 h before day 1 of the EMA phase.

### MRI acquisition

Brain imaging data were collected using a 3.0 Tesla GE MRI system equipped with a 32-channel head coil. Anatomical volumes were acquired using a sagittal 3D T1-weighted sequence (Repetition Time = 8.5 ms, Echo Time = 3.2 ms, flip angle = 11°, FOV = 256 mm × 256 mm, voxel size = 1 mm^3^, 176 slices). The resting-state functional images were collected using a single-shot echo-planar sequence (RT = 2.2 s, ET = 27 ms, flip angle = 80°, FOV = 192 mm × 192 mm, voxel size = 3 mm × 3 mm × 3.5 mm, 42 axial slices), during which participants were instructed to keep their eyes closed, remain awake, and not think about anything in particular.

### Preprocessing

Preprocessing steps included bias field correction, skull stripping, MNI normalization, segmentation, slice timing, motion correction, distortion correction, coregistration, regression of motion parameters, and band pass filtering were conducted using FMRIPREP [34]. Further details can be found in [35]. All MRI were inspected by a radiologist for anatomical anomalies that could interfere with image analysis and resulted in the exclusion of 4 controls and 2 patients with SUD; additionally, 2 patients with SUD and 1 control with movement >3 mm during the rs-fMRI acquisition were excluded from the rs analysis. First level rs analyses including network computation, ROI extraction, and individual connectome calculation were conducted using Nilearn [36] on python 3.9 [37]. Second level connectivity analyses were carried out using the CONN.16 toolbox [38] implemented in SPM12 Software (https://www .fil.ion.ucl.ac.uk/spm/software/spm12/) on MATLAB, version 12.0 (http:// www.mathworks.fr/products/matlab).

### Dictionary learning assessments of functional connectivity

Dictionary Learning is a multivariate reduction method allowing decomposition of 4D rs-brain signals into a set of spatially located functional networks. In contrast to an Independent Component Analysis (ICA), decomposition is based on sparsity rather than independence by combining a sparsity-inducing penalty to a reconstruction loss, resulting in better performance, stability, and higher contrast than ICA (for details see [39]). Based on current recommendations in the literature and data driven estimation of number of components derived from MELODIC version 3.04 [40], we extracted 20 components of the signal. Resulting spatial maps were then reduced to 84 regions of interest (ROI) by keeping the more intense nonzero brain voxels across all maps with a threshold of 0.5. Individual connectomes were then computed using Pearson correlations between BOLD signals in all ROIs (for Dictionary Learning analyses, see: https://nilearn.github.io/stable/connectivity/resting_state_networks.html). The MNI coordinates of each ROI’s center were extracted and automatically labeled using label4MRI (retrieved from: https://github.com/yunshiuan/label4MRI) on R [32].

### Statistical analyses

#### Group comparisons

Due to the non-normality of the data distribution, group comparisons for Stroop performance, substance use, and craving were performed using non-parametric tests: Mann-Whitney tests for the effect of group (patients versus controls) and one-way Kruskal–Wallis (non-parametric) ANOVA and post-hoc Dwass-Steel-Critchlow-Fligner (DSCF) pairwise comparisons for the effect of SUD sub-groups (alcohol, tobacco, cannabis).

#### Hierarchical modeling of craving, Inhibition functioning, and substance use

We used the lmer4 packages available on R [41] to fit the generalized linear mixed-effects model (for binomial outcomes) and Linear Mixed-Effects Models (for continuous outcomes) with random intercepts on the EMA sample (presented in supplementary Table 1). We modeled a time lag in our measures to predict an outcome at time t + 1 from any variables at time t. To avoid contamination of night-time effects, this time lag excluded all predictions of the first assessment of a new day by the last assessment of the previous days. Missing data in either the outcome or the predictor were discarded from the analysis.

Craving level was centered around the participant’s habitual level during the week. To take into account both within and between-person level variations in Stroop performances we used two centering methods [42]. Individual-centered Stroop refers to the momentary performance centered around the participant’s own level during the week, hence depicting momentary increase or decrease in inhibition performance. Group-centered Stroop refers to the momentary performance centered around the SUD group level during the week to further take into account high or low momentary performances. Use of any substance, use of primary substance, and Stroop performances at time t were successively entered as predictors of use of any substance, use of primary substance, and Stroop performances at time t + 1. For description purposes, we also assessed the impact of time defined as the number of assessment occasions on substance use. To test for a moderating effect of the Stroop on the craving/substance use link, we entered the interactions term craving * Stroop performance at time t as the predictor of substance use at time t + 1. To assess potential learning effects, we also tested the time * Stroop performances interaction impact on substance use at time t + 1. Finally, we conducted Causal Mediations Analysis using the mediation package available on R [43] to further characterize the interplay between and among all the significant main effects.

#### Whole brain ROI to ROI functional connectivity

To assess resting-state networks related to executive functioning in the SUD group of the MRI sample, we employed user-defined contrast in the Conn Toolbox with Stroop time as the between-subject variable and the connectivity strength in the connectome derived from Dictionary Learning as the outcome variable while controlling for age. The results were corrected for multiple comparisons using false discovery rate (FDR) with an alpha level of 0.05.

The strength of connections in the identified networks were then extracted in the two groups (SUD and control) for comparison. Finally, Spearman correlations were used to assess relations between connectivity strength and the sum of substance use occasions during the week for any substance and for the primary substances in the SUD group. These analyses were performed using JAMOVI software version 2.3 (The jamovi project (2022). Retrieved from https://www.jamovi.org). All statistical tests were two-sided and results were considered significant when *p* ≤ 0.05 was reached.

## Results

### Sample characteristics

All behavioral analyses were based on the overall EMA sample of 99 participants: 64 individuals with SUD (33 men; mean age 41.66 ± 11.81), including 32 treated for alcohol, 20 for tobacco, and 12 for cannabis use disorder. The overall prevalence of comorbid depression was 17.2%. The control group included 35 participants (18 men; mean age 34 ± 8.22). Supplementary Table 1 presents the descriptive statistics of these groups. The neuroimaging analyses included the MRI sample, consisting of 74 of the 99 EMA participants: 40 SUD patients (20 men; mean age 42.42 ± 11.34), including 20 treated for alcohol, 13 for tobacco and 7 for cannabis use disorders. The overall prevalence of comorbid depression in the SUD group was 12.5%. The control group included 34 participants (18 men; mean age 34.26 ± 8.19). Statistical results for descriptive and group comparisons are presented in Table 1 for the MRI sample.

**Table 1:** Descriptive statistics of the behavioral and psychological variables of interest in the MRI sample (*n* = 74).

	Healthy control (*N* = 34)			Any substance (*N* = 40)			Alcohol (*N* = 20)			Tobacco (*N* = 13)			Cannabis (*N* = 7)		
	M	SD	%	M	SD	%	M	SD	%	M	SD	%	M	SD	%
Age	34.26^**^	8.19		42.4	11.30		43.00^B^	10.60		47.30^C^	9.07		31.70	11.50	
Sex (% male)			52.94			50.00			65.00			23.07			57.14
Education (years)	14.40^*^	2.95		13.10	2.29		13.30	2.23		13.90^C^	2.22		11.10	1.57	
Current depressive comorbidity			-			14.70			20.00			7.69			0.00
Compliance			94.29^***^			86.29			87.43			86.29			83.14
Addiction severity for primary Substance (ASI)	-	-		6.45	0.75		6.50	0.69		6.31	0.86		6.57	0.79	
Mean stroop time (s)	10.74^***^	2.24		14.28^M^	4.69		15.43	5.50		13.06	3.32		12.55	3.79	
Mean craving (per day)	1.02^***^	0.05		2.87 ^F^	1.32		2.60	0.93		2.62	1.40		4.09	1.60	
Substance use of any substance (Per day)	0.29^***^	0.61		3.13^M^	1.55		3.44^A^	1.53		2.68	1.52		3.10	1.46	
Substance use of primary substance (per day)	-	-		1.81^F^	1.50		1.40^A^	1.39		2.44^C^	1.51		1.80	1.40	

*S.d.* standard deviation, *Min* minimum, *Max* maximum. *<0.05; **<0.01; ***<0.001, *A* Alcohol ≠ Tobacco, *B* Alcohol ≠ Cannabis; C Tobacco ≠ Cannabis. *M* Male (patient) > Female (patient), *F* Female (patient) > Male (patient).

### Association of craving, substance use, and executive functioning in SUD

Statistical results for principal and mediations effects in the SUD group are presented in Table 2a–c. Predictions include the two operationalization of Stroop performance, group-centered and individual-centered.

**Table. 2:** Results of the substance use as outcomes models in the EMA sample.

a. Prediction of substance use at time t+1, by precedent use and Stroop time (individual or group-centered) levels									
			Outcome at time *t* + 1: use of						
	Primary substance					Any substance			
Predictors at time *t*	*γ*	SE		*Z* value	*p* value	γ	SE	*Z* value	*p* value
Any substance use	0.5612	0.2071		2.709	<0.01^A^	0.6775	0.20050	3.379	<0.001^A^
Primary substance use	0.7650	0.1725		4.434	<0.001^A^	0.2525	0.21050	1.200	0.23
Craving	0.3632	0.0763		4.763	<0.001^A^	0.3897	0.0902	4.319	<0.001^A^
Stroop time (individual-centered)	−0.1189	0.1346		−0.883	0.38	−0.3295	0.1549	−2.127	0.03^C^
Stroop time (group-centered)	0.3558	0.1813		1.963	0.049^B^	0.2768	0.284203	0.974	0.33
Stroop × craving	0.0715	0.1349		−0.530	0.60	−0.1501	0.1852	−0.810	0.42
Stroop × time	0.0306	0.0164		1.867	0.06	0.0046	0.017933	0.251	0.80

b. Prediction of Stroop time (individual or group-centered) by precedent substance use								
	Predictors at time *t*: use of							
	Primary substance				Any substance			
Outcome at time *t* + 1	γ	SE	*Z* value	*p* value	*γ*	SE	Z value	*p* value
Stroop time (individual-centered)	0.3601	0.1316	2.737	<0.01^A^	0.2267	0.1486	1.526	0.13
Stroop time (group-centered)	0.7625	0.2198	3.470	<0.001^A^	0.7546	0.3317	2.275	0.02

c. Prediction of primary substance use at time t+1 by use of primary substance at time t while entering Stroop time at time t as mediator				
	Outcome at time *t* + 1: use of primary substance			
Effect type by predictors at time *t*	Estimate	Lower CI	Upper CI	*p* value
Average causal mediation effects by Stroop time (group-centered)	0.0167	0.0023	0.04	<0.02
Average direct effects of substance use	0.2131	0.1105	0.31	<0.001
Total effect	0.2298	0.1300	0.32	<0.001
Proportion mediated by Stroop time (group-centered)	0.0624	0.0094	0.19	0.02

^A^Surviving correction for age, sex, education, addiction severity, and primary substance type.

^B^Surviving correction for previous use at time *t*, craving at time t, sex, education, addiction severity, and primary substance type but not for age.

^C^Surviving correction for previous use at time t, age, sex, education, addiction severity, and primary substance type but not for craving at time *t.*

*CI* confidence interval at 95%.

#### Prediction of substance use at t + 1 by use at time t (Table 2a)

Use of any substance at a given time t significantly predicted later use of both any or primary substance. Use of primary substance only predicted later use of primary substances but not the use of any substances.

#### Prediction of use at t + 1 by Stroop at time t (Table 2a)

Increase in Individual-centered Stroop performance at time t significantly predicted a lower probability of the use of any substance at time t + 1. Better Group-centered Stroop performance at time t significantly predicted the use of the primary substance at time t + 1 but not the use any substance.

#### Prediction of use at t + 1 by craving at time t (Table 2a)

Increase in craving at time t significantly predicted a higher probability of the use of any substance and of the primary substance at time t + 1.

#### Prediction of Stroop scores at t + 1 by use at time t (Table 2b)

Decrease in Individual-centered Stroop performance at time t + 1 was significantly predicted by use of the primary substance at time t but not by use of any substance. Worst Group-centered Stroop performance at time t + 1 was significantly predicted by both the use of the treated and any substance at time t.

#### Interaction of Stroop * craving on substance use (Table 2a)

The Stroop * craving interaction at time t did not predict significantly the use of any or primary substance at time t + 1.

#### Interaction of Stroop * time on substance use (learning effect) (Table 2a)

Our results indicated no significant effect on Stroop * time interaction at time t and use of any or primary substance at time t + 1.

#### Mediation of Stroop between use at time t and use at time t + 1 (Table 2c)

Group-centered Stroop performance at time t significantly mediated the relationship between use of primary substance at time t and use of primary substance at time t + 1 (see Supplementary Figure 2).

### Neuroimaging results

#### Dictionary learning networks and ROI extraction

We computed 20 Dictionary Learning components, representing resting state networks. Graphical description components and their corresponding variance explained appear in supplementary Figure 1. From these components, 84 regions of interest were extracted. Description of each ROI including their center MNI coordinates and labels according to the AAL atlas are in Supplementary Table 2.

#### Stroop time-related connectivity in the SUD group

**Fig. 2:**
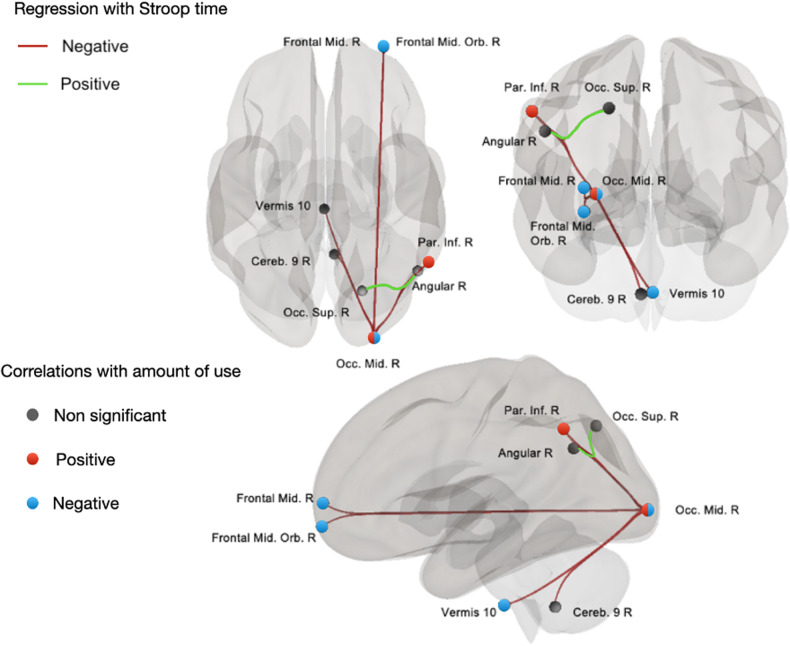
Rs connections linked to mean stroop time and amount of substance use during the week. Cereb Cerebellum, Occ. Occipital, Par. Parietal, Mid. Middle, Sup. Superior, Inf. Inferior, Orb. Orbital, R Right. Regression analyses were conducted between the individual connectomes and mean Stroop times from each participant while controlling for age and correcting for false discovery rate (FDR) with alpha level of 0.05. Resulting connections are highlighted in the above figure and colored depending on their association with Stroop times, positively (highlighted in green) or negatively (highlighted in red). The strength of connectivity within these connections were entered in a correlation matrix along with the number of any or primary substance use occasions. Significant correlations with use are highlighted by the node colored in red (positive correlation) and blue (for negative correlation).

The mean Stroop time across all tests correlated with the strength of connectivity within 7 pairs of areas in the SUD group while controlling for age (see Fig. 2). Longer Stroop times correlated with greater strength of connectivity between the right angular and right superior occipital area. Further, longer Stroop times correlated with weaker connectivity between the right middle occipital area and five right unilateral and one midline region: the interior parietal, middle orbital frontal, middle frontal, angular, cerebellar lobule IX, and vermis 10.

#### Associations between rs-MR functional networks and EMA variables

No significant difference was found between the SUD and the control groups in connectivity strength in the 7 above-mentioned connections (See Table 3). Moreover, significant negative correlations were found between the number of any substance use occasions during the week and connectivity strength between the right middle occipital and cerebellar vermis 10 areas and the middle frontal and right orbital frontal areas in the SUD group. Finally, a greater number of primary substance use occasions correlated with stronger connectivity between the right middle occipital and right inferior parietal areas and are presented in Table 4 and Fig. 2.

**Table 3:** Non parametric group comparisons of rs networks connectivity strength between the SUD and the control group.

	Occ. Mid. R-Frontal Mid. R	Occ. Mid. R-AngularR	Occ. Mid. R-Vermis10	Occ. Mid. R-Frontal Mid. Orb. R	Occ. Mid. R-Par. Inf. R	Occ. Sup. R-AngularR	Occ. Mid. R – Cereb. 9 R
Rank-biserial	0.035	0.025	0.078	0.206	0.082	0.131	0.029
*P* value	0.800	0.859	0.571	0.131	0.549	0.339	0.833

*Occ.* occipital, *Par*. parietal, *Mid*. middle, *Sup*. superior, *Inf*. inferior, *Orb.* orbital, *R* right, *Cereb*. cerebellum.

**Table 4:** Correlations between number of substances use occasions within the week and rs networks connectivity in the SUD group.

		Occ. Mid. R-Frontal Mid. R	Occ. Mid. R-AngularR	Occ. Mid. R-Vermis10	Occ. Mid. R-Frontal Mid. Orb. R	Occ. Mid. R-Par. Inf. R	Occ. Sup. R-AngularR	Occ. Mid. R – Cereb. 9 R
Use of any substance	Spearman rho	**−0.336**	−0.205	**−0.323**	**−0.363**	−0.104	0.201	−0.178
	*P* value	**0.034**	0.205	**0.042**	**0.021**	0.522	0.213	0.062
Use of primary substance	Spearman rho	0.144	0.011	−0.062	0.074	**0.339**	0.049	−0.071
	*P* value	0.377	0.948	0.704	0.651	**0.032**	0.766	0.664

*Occ.* occipital, *Par*. parietal, *Mid*. middle, *Sup*. superior, *Inf*. inferior, *Orb.* orbital, *R* right, *Cereb*. cerebellum.

Bold values highlight significant results.

## Discussion

To the best of our knowledge, we characterized for the first time the time course of daily use in interaction with inhibition function in patients under treatment for primary alcohol, tobacco, and cannabis use disorders. Not only did use exhibit a different pattern regarding primary or any substance, but the contribution of inhibition in averting use also differed for both outcomes, whereby the protective role was beneficial only on the primary drug during treatment. Although this effect was not explained by an interaction with craving, it was partially sustained by rs-connectivity strength in frontal, occipital, parietal, and cerebellar areas, itself being linked to the frequency of use.

During a typical day of EMA, the use of any substance predicted increased probabilities of use of both any and the primary substance during the following hour. Good inhibition performance, indicated by the performance at the time of assessment in comparison to the group, neither interacted with this pattern nor conferred protection from the use of any substance. Conversely, the use of the primary substance predicted only an increased probability of use of this same substance during the following hours and was impacted by better momentary inhibition performance than the group that directly predicted lower probabilities of later use for the primary substance. Moreover, differences in inhibitory control mediated this pattern of use, where better inhibitory control enabled one to resist the urge to use the same substance again, and where compromised inhibitory control precipitated substance use. This study is not the first to demonstrate higher risk use of a primary substance after having used another substance [44, 45] but suggests that once the primary substance is used, patients tend to use this substance again rather than a different one.

The influence of the inhibition performance on use was also dependent of previous use. A sudden drop in inhibition performance, as indicated by the performance of this assessment compared to the patient’s own mean, predicted a lower probability of later use of any substance. Whereas poorer inhibition performance as predicting a lower probability of later use could seem surprising, it was predicted by the use of primary substance within the previous hours. This study thus replicated the already described deleterious effect of use on later inhibition performance, for alcohol [46], cannabis [47], although such effects in tobacco use are an ongoing debate [48].

Concerning how inhibition integrity protects one from relapse, our results supported certain hypotheses regarding the protective effect of brain integrity. Our brain imaging analysis revealed that better inhibition performance was sustained by specific connections that did not significantly differ between patients with SUD and controls. Better Stroop performance was linked to stronger connectivity between the occipital cortex and parietal, frontal, and cerebellar areas and to weaker connectivity between the right angular and the right occipital superior areas. Such brain regions have been linked to inhibition functions during the Stroop interference task in healthy populations with activations studies citing the involvement of the right middle occipital gyrus, right middle and orbital frontal areas, right parietal inferior areas [49], angular gyrus [50] and the cerebellum [51]. Meta-analytic studies applying conjunction analysis to executive functioning tasks emphasize the involvement of these areas in a more global, superordinate network that would support cognitive control [52]. Specific regional engagement within this network could vary, however, to adapt to task-specific demands allowing for stronger frontal connectivity with sensory or motor regions, such as the cerebellum, to support response selection and inhibition [53]. As consequences, the observed protective effect of inhibition could be mediated by cerebral integrity within those networks as previously suggested [9, 54–56].

Inhibition performance could also be interpreted as protecting one from substance use by moderating the effect of craving. In support of this interpretation, we previously demonstrated that executive-related brain areas were involved in the regulation of craving dynamics over time [13]. Even if both craving and inhibition independently predicted use, our current study failed to observe significant interactions between craving and inhibition performance, thus precluding endorsement of this interpretation.

Although patients with SUD performed worse at the mobile Stroop task than the control group, the highest functioning patients with SUD compared to their own group exhibited the lowest tendencies to use their primary substance, better ability to resist even after an episode of use, and the highest functional connectivity integrity in Stroop-related networks. These protective effects were closely linked to the participants’ preferred substance. These results could be interpreted in light of the Limited Resource theory of self-control that posits that the high cognitive demands imposed by self-control over impulses (such as trying to refrain from using a substance) can lead to a depletion state, namely ‘ego depletion’, of this resource [57] and thus in the case of SUD higher risk of substance use [14]. It would be possible that in a similar manner, patients direct high self-control towards the substance they are supposed to avoid, leading to lower allocations of the inhibition component toward the avoidance of other substances. In line with recent recommendations, our work emphasizes the potential of targeting inhibition in populations treated for different types of SUD [21] but raises important considerations regarding its limits as they apply to polysubstance use.

### Limitation

Several limits should be considered when interpreting the results of the present study. Firstly, because our research focused on the dissociation between the primary substance during treatments versus any substances, we did not assess the availability of the primary drug in the immediate environment. It is thus possible that the use of any substance could reflect momentary preference or more simply unavailability of the primary drug. Secondly, craving assessments were not specific to the primary substance but general, which could impact the interaction between craving and other risk factors. Thirdly, should be noted that for the feasibility of EMA settings, the Stroop-like mobile test only includes the incongruent condition without the baseline control condition. While this standard in EMA settings has already been linked to accurate assessments of executive functioning in patients with SUD [33, 35], potential effects of visuo-motor speed confounds may be present in these data. Finally, whereas we focused our article on inhibition, others cognitive components can interplay during the Stroop test. Indeed, attentional components, particularly attention to the relevant stimuli, could also play an important role in our results. Further studies could be conducted to uncover the unique and joint contribution of diverse executive and attention processes in use.

## Conclusion

Inhibitory control is a potentially powerful target of intervention, enabling the opportunity to break the precipitating relapse cycle that is characteristic of SUD treatment attempts. This beneficial effect, however, may be restricted to the primary drug during treatment, whereas polysubstance use loom as a critical risk factor in clinical settings.

### Supplementary information

Supplementary Materials

## Data Availability

Data are available upon request to the corresponding author.

## References

[1] Grant BF, Goldstein RB, Saha TD, Chou SP, Jung J, Zhang H (2015). Epidemiology of DSM-5 alcohol use disorder: results from the national epidemiologic survey on alcohol and related conditions III. JAMA Psychiatry.

[2] Merikangas KR, He J, Burstein M, Swanson SA, Avenevoli S, Cui L et al. Lifetime prevalence of mental disorders in U.S. adolescents: results from the national comorbidity survey replication–adolescent supplement (NCS-A). J Am Acad Child Adolesc Psychiatry. 2010;49:980–9. 10.1016/j.jaac.2010.05.017 PMC2946114 20855043

[3] Sacks JJ, Gonzales KR, Bouchery EE, Tomedi LE, Brewer RD (2015). 2010 national and state costs of excessive alcohol consumption. Am J Prev Med.

[4] Tiffany ST, Friedman L, Greenfield SF, Hasin DS, Jackson R (2012). Beyond drug use: a systematic consideration of other outcomes in evaluations of treatments for substance use disorders: beyond drug use. Addiction.

[5] Shoptaw S, Commentary on Gowin. (2014). (2014): Brain is behavior-methamphetamine dependence and recovery: commentary. Addiction.

[6] Domínguez-Salas S, Díaz-Batanero C, Lozano-Rojas OM, Verdejo-García A (2016). Impact of general cognition and executive function deficits on addiction treatment outcomes: systematic review and discussion of neurocognitive pathways. Neurosci Biobehav Rev.

[7] Miyake A, Friedman NP, Emerson MJ, Witzki AH, Howerter A, Wager TD (2000). The unity and diversity of executive functions and their contributions to complex “frontal lobe” tasks: a latent variable analysis. Cogn Psychol.

[8] Lozano-Rojas ÓM, Gómez-Bujedo J, Pérez-Moreno PJ et al. Impulsivity Predicts Relapse—but Not Dropout—in Outpatients with SUD: a Longitudinal Study. Int J Ment Health Addiction 2023. 10.1007/s11469-023-01024-y.

[9] Everitt BJ (2014). Neural and psychological mechanisms underlying compulsive drug seeking habits and drug memories–indications for novel treatments of addiction. Eur J Neurosci.

[10] Jakubiec L, Chirokoff V, Abdallah M, Sanz-Arigita E, Dupuy M, Swendsen J (2022). The executive functioning paradox in substance use disorders. Biomedicines.

[11] Barocas JA, Wang J, Marshall BDL, LaRochelle MR, Bettano A, Bernson D (2019). Sociodemographic factors and social determinants associated with toxicology confirmed polysubstance opioid-related deaths. Drug Alcohol Depend.

[12] Lukasiewicz M, Fareng M, Benyamina A, Blecha L, Reynaud M, Falissard B (2007). Ecological momentary assessment in addiction. Expert Rev Neurother.

[13] Chirokoff V, Abdallah M, Chanraud S, J Swendsen. Connectivity of cortico-cerebellar loops at rest underlies craving instability and predict consumption in the addict population. 2022. 10.13140/RG.2.2.29115.28963.

[14] Jones A, Christiansen P, Nederkoorn C, Houben K, Field M. Fluctuating disinhibition: implications for the understanding and treatment of alcohol and other substance use disorders. Front Psychiatry 2013;4:140. 10.3389/fpsyt.2013.00140 PMC3804868 24155728

[15] Flaudias V, Heeren A, Brousse G, Maurage P (2019). Toward a triadic approach to craving in addictive disorders: the metacognitive hub model. Harv Rev Psychiatry.

[16] Goldstein RZ, Volkow ND (2002). Drug addiction and its underlying neurobiological basis: neuroimaging evidence for the involvement of the frontal cortex. Am J Psychiatry.

[17] Kreek MJ, Nielsen DA, Butelman ER, LaForge KS (2005). Genetic influences on impulsivity, risk taking, stress responsivity and vulnerability to drug abuse and addiction. Nat Neurosci.

[18] Cadet JL, Bisagno V, Milroy CM (2014). Neuropathology of substance use disorders. Acta Neuropathol (Berl).

[19] Lundqvist T. Imaging cognitive deficits in drug abuse. In: Self DW, Staley Gottschalk JK (eds). Behavioral neuroscience of drug addiction. Springer: Berlin, Heidelberg, 2010; 247–75. 10.1007/7854_2009_26 21161756

[20] Yücel M, Lubman DI, Solowij N, Brewer WJ (2007). Understanding drug addiction: a neuropsychological perspective. Aust N Z J Psychiatry.

[21] Kwako LE, Momenan R, Litten RZ, Koob GF, Goldman D (2016). Addictions neuroclinical assessment: a neuroscience-based framework for addictive disorders. Biol Psychiatry.

[22] Schmidt TP, Pennington DL, Cardoos SL, Durazzo TC, Meyerhoff DJ (2017). Neurocognition and inhibitory control in polysubstance use disorders: comparison with alcohol use disorders and changes with abstinence. J Clin Exp Neuropsychol.

[23] Parvaz MA, Rabin RA, Adams F, Goldstein RZ (2022). Structural and functional brain recovery in individuals with substance use disorders during abstinence: a review of longitudinal neuroimaging studies. Drug Alcohol Depend.

[24] Stevens L, Verdejo-García A, Goudriaan AE, Roeyers H, Dom G, Vanderplasschen W (2014). Impulsivity as a vulnerability factor for poor addiction treatment outcomes: A review of neurocognitive findings among individuals with substance use disorders. J Subst Abus Treat.

[25] Han B, Compton WM, Blanco C, DuPont RL (2017). National trends in substance use and use disorders among youth. J Am Acad Child Adolesc Psychiatry.

[26] Staiger PK, Richardson B, Long CM, Carr V, Marlatt GA (2013). Overlooked and underestimated? Problematic alcohol use in clients recovering from drug dependence: The impact of alcohol on drug treatment outcomes. Addiction.

[27] Serre F, Fatseas M, Swendsen J, Auriacombe M (2015). Ecological momentary assessment in the investigation of craving and substance use in daily life: a systematic review. Drug Alcohol Depend.

[28] Brisseau S, Auriacombe M, Franques P, Daulouède J-P, Tignol J (1999). L’addiction severity index. Courr Addict.

[29] McLellan AT, Kushner H, Metzger D, Peters R, Smith I, Grissom G (1992). The fifth edition of the addiction severity index. J Subst Abus Treat.

[30] Denis C, Fatséas M, Beltran V, Serre F, Alexandre J-M, Debrabant R (2016). Usefulness and validity of the modified addiction severity index: a focus on alcohol, drugs, tobacco, and gambling. Subst Abus.

[31] Sheehan DV, Lecrubier Y, Sheehan KH, Amorim P, Janavs J, Weiller E (1998). The Mini-International Neuropsychiatric Interview (M.I.N.I.): the development and validation of a structured diagnostic psychiatric interview for DSM-IV and ICD-10. J Clin Psychiatry.

[32] R Core Team. R: a language and environment for statistical computing. R foundation for statistical computing: Vienna, Austria, 2020 https://www.R-project.org/.

[33] Bouvard A, Dupuy M, Schweitzer P, Revranche M, Fatseas M, Serre F (2018). Feasibility and validity of mobile cognitive testing in patients with substance use disorders and healthy controls. Am J Addict.

[34] Esteban O, Blair R, Markiewicz CJ, Berleant SL, Moodie C, Ma F et al. poldracklab/fmriprep: 1.0.0-rc5. 2017. 10.5281/zenodo.996169.

[35] Dupuy M, Abdallah M, Swendsen J, N’Kaoua B, Chanraud S, Schweitzer P (2022). Real-time cognitive performance and positive symptom expression in schizophrenia. Eur Arch Psychiatry Clin Neurosci.

[36] Abraham A, Pedregosa F, Eickenberg M, Gervais P, Mueller A, Kossaifi J et al. Machine learning for neuroimaging with scikit-learn. Front Neuroinform. 2014;8:14. 10.3389/fninf.2014.00014 PMC3930868 24600388

[37] Van Rossum G, Drake FL. Python 3 reference manual. CreateSpace: Scotts Valley, CA, 2009.

[38] Whitfield-Gabrieli S, Nieto-Castanon A (2012). *Conn*: a functional connectivity toolbox for correlated and anticorrelated brain networks. Brain Connect.

[39] Mensch A, Varoquaux G, Thirion B. Compressed online dictionary learning for fast resting-state fMRI decomposition. In: International symposium on biomedical imaging. IEEE: Prague, Czech Republic, 2016, pp 1282–5.

[40] Beckmann CF, Smith SM (2004). Probabilistic independent component analysis for functional magnetic resonance imaging. IEEE Trans Med Imaging.

[41] Bates D, Mächler M, Bolker B, Walker S (2015). Fitting linear mixed-effects models using lme4. J Stat Softw.

[42] Kreft IGG, De Leeuw J, Aiken LS (1995). The effect of different forms of centering in hierarchical linear models. Multivar Behav Res.

[43] Tingley D, Yamamoto T, Hirose K, Keele L, Imai K (2014). mediation: R package for causal mediation analysis. J Stat Softw.

[44] Preston KL, Jobes ML, Phillips KA, Epstein DH (2016). Real-time assessment of alcohol drinking and drug use in opioid-dependent polydrug users. Behav Pharm.

[45] Epstein DH, Marrone GF, Heishman SJ, Schmittner J, Preston KL (2010). Tobacco, cocaine, and heroin: craving and use during daily life. Addict Behav.

[46] Field M, Wiers RW, Christiansen P, Fillmore MT, Verster JC. Acute alcohol effects on inhibitory control and implicit cognition: implications for loss of control over drinking: acute alcohol effects on cognitive function. Alcohol Clin Exp Res. 2010;34:1346–1352. 10.1111/j.1530-0277.2010.01218.x PMC2999764 20491732

[47] Oomen PP, van Hell HH, Bossong MG (2018). The acute effects of cannabis on human executive function. Behav Pharm.

[48] Newhouse P (2004). Effects of nicotinic stimulation on cognitive performance. Curr Opin Pharm.

[49] Song Y, Hakoda Y (2015). An fMRI study of the functional mechanisms of Stroop/reverse-Stroop effects. Behav Brain Res.

[50] Coderre EL, van Heuven WJB (2013). Modulations of the executive control network by stimulus onset asynchrony in a Stroop task. BMC Neurosci.

[51] Okayasu M, Inukai T, Tanaka D, Tsumura K, Hosono M, Shintaki R et al. An excitatory-inhibitory fronto-cerebellar loop resolves the Stroop effect. 2022; https://www.biorxiv.org/content/10.1101/2022.01.18.476551v1. 10.1038/s41467-022-35397-w PMC9834394 36631460

[52] Niendam TA, Laird AR, Ray KL, Dean YM, Glahn DC, Carter CS (2012). Meta-analytic evidence for a superordinate cognitive control network subserving diverse executive functions. Cogn Affect Behav Neurosci.

[53] Bellebaum C, Daum I (2007). Cerebellar involvement in executive control. Cerebellum.

[54] Adinoff B (2004). Neurobiologic Processes in Drug Reward and Addiction. Harvard Review of Psychiatry.

[55] Jentsch JD, Taylor JR (1999). Impulsivity resulting from frontostriatal dysfunction in drug abuse: implications for the control of behavior by reward-related stimuli. Psychopharmacology.

[56] Lyvers M (2000) “Loss of control” in alcoholism and drug addiction: A neuroscientific interpretation. Experimental and Clinical Psychopharmacology. 2000;8:225–45. 10.1037//1064-1297.8.2.225 10843306

[57] Baumeister RF, Muraven M, Tice DM (2000). Ego depletion: a resource model of volition, self-regulation, and controlled processing. Soc Cogn.

