# Supplementary material for: Identifying the role of (dis)inhibition in the vicious cycle of substance use through ecological momentary assessment and resting-state fMRI
Source: Transl Psychiatry. 2024 Jun 19;14:260. doi: 10.1038/s41398-024-02949-1 (PMC11186821; doi:10.1038/s41398-024-02949-1)
Supplement: Supplementary file 1 — Supplementary Materials [file 41398_2024_2949_MOESM1_ESM.docx]

**SUPPLEMENTARY TABLES**

**Supplementary table 1**: Descriptive statistics of the behavioral and psychological variables of interest in the EMA sample (n= 99)

|  | Healthy Control (N=35) | | | Any SUD (N=64) | | | Alcohol (N= 32) | | | Tobacco (N=20) | | | Cannabis (N=12) | | |
| --- | --- | --- | --- | --- | --- | --- | --- | --- | --- | --- | --- | --- | --- | --- | --- |
|  | M | SD | % | M | SD | % | M | SD | % | M | SD | % | M | SD | % |
| Age | 34 ^*^ | 8.22 |  | 41.66 | 11.81 |  | 44.20 ^B^ | 11.30 |  | 43.40 ^C^ | 11.10 |  | 32.00 | 10.20 |  |
| Sex (% male) |  |  | 51.42 |  |  | 51.56 |  |  | 62.50 ^B^ |  |  | 20 ^C^ |  |  | 81.81 |
| Education (years) | 14.40 | 2.91 |  | 13.40 | 2.52 |  | 13.10 | 2.24 |  | 14.40 | 2.70 |  | 12.60 | 2.57 |  |
| Current Depressive Comorbidity |  |  | - |  |  | 17.18 |  |  | 21.87 |  |  | 5.00 |  |  | 27.27 |
| Compliance |  |  | 94.29^***^ |  |  | 86 |  |  | 87.43 |  |  | 86 |  |  | 83.43 |
| Addiction Severity for primary Substance (ASI) | - | - |  | 6.52 | 0.73 |  | 6.53 | 0.76 |  | 6.45 | 0.76 |  | 6.58 | 0.67 |  |
| Mean Stroop Time (s) | 10.67^***^ | 2.248 |  | 14.43 | 4.91 |  | 16.07 ^A^ | 5.88 |  | 12.88 | 2.91 |  | 12.61 ^B^ | 3.12 |  |
| Mean craving (Per day) | 1.02^***^ | 0.10 |  | 2.85 | 1.46 |  | 2.51 ^B^ | 0.971 |  | 2.87 | 1.40 |  | 3.73 | 1.47 |  |
| Substance use of any substance (Per day) | 0.28^***^ | 0.61 |  | 3.20 | 1.58 |  | 3.48 | 1.52 |  | 2.89 | 1.61 |  | 2.98 | 1.54 |  |
| Substance use of primary substance (Per day) | - | - |  | 1.98 | 1.66 |  | 1.52 ^A^ | 1.53 |  | 2.73 | 1.62 |  | 1.92 | 1.62 |  |

S.d. : standard deviation; Min : minimum; Max. : maximum ;* : <.05; **< .01 ; ***<.001 ; A : Alcohol ≠ Tobacco ; B : Alcohol ≠ Cannabis ; C Tobacco ≠ Cannabis.

**Supplementary Table 2:** Description of the MNI coordinates and AAL label for the center of the 84 Regions Of Interest derived from the Dictionary Learning networks.

| **ROI** | **MNI_x** | **MNI_y** | **MNI_z** | **AAL_label** |
| --- | --- | --- | --- | --- |
| **1** | -8,58 | -30,44 | 62,89 | Paracentral_Lobule_L |
| **2** | -43,17 | 4,41 | -16,23 | Temporal_Pole_Sup_L |
| **3** | -0,43 | -28,59 | -47,63 | Vermis_10 |
| **4** | 9,08 | -22,01 | 13,82 | Thalamus_R |
| **5** | 43,45 | 8,71 | -17,35 | Temporal_Pole_Sup_R |
| **6** | -36,44 | 17,85 | -2,31 | Insula_L |
| **7** | 44,79 | 19,73 | 1,49 | Frontal_Inf_Oper_R |
| **8** | -29,45 | -75,62 | -24,19 | Cerebelum_Crus1_L |
| **9** | -32,02 | 60,47 | 6,52 | Frontal_Mid_L |
| **10** | 0,92 | -12,53 | 10,97 | Thalamus_R |
| **11** | 5,85 | 9,25 | 70,80 | Supp_Motor_Area_R |
| **12** | 26,61 | -79,18 | -23,43 | Cerebelum_Crus1_R |
| **13** | 32,82 | 61,96 | 3,47 | Frontal_Mid_R |
| **14** | -52,57 | -20,76 | 27,89 | Postcentral_L |
| **15** | 31,44 | 18,96 | 18,53 | Insula_R |
| **16** | 51,04 | -42,35 | 25,16 | SupraMarginal_R |
| **17** | -57,27 | -26,29 | -17,47 | Temporal_Inf_L |
| **18** | 2,62 | 38,56 | -16,38 | Rectus_R |
| **19** | 56,78 | -10,91 | -21,22 | Temporal_Mid_R |
| **20** | -13,75 | 21,08 | 26,76 | Cingulum_Ant_L |
| **21** | 5,68 | -41,58 | -63,60 | Cerebelum_9_R |
| **22** | 4,48 | -29,26 | -4,94 | Lingual_R |
| **23** | 9,63 | 18,50 | 27,25 | Cingulum_Ant_R |
| **24** | -42,60 | 0,11 | 37,82 | Precentral_L |
| **25** | -39,44 | 39,55 | 36,76 | Frontal_Mid_L |
| **26** | 28,55 | -64,93 | 48,68 | Angular_R |
| **27** | 40,60 | 2,47 | 42,40 | Precentral_R |
| **28** | -60,29 | -19,73 | 10,01 | Temporal_Sup_L |
| **29** | -33,42 | -81,34 | -13,83 | Fusiform_L |
| **30** | -30,74 | -43,17 | -54,09 | Cerebelum_8_L |
| **31** | -31,94 | 4,24 | -18,12 | Amygdala_L |
| **32** | -9,95 | -71,36 | 61,23 | Precuneus_L |
| **33** | -6,68 | -40,36 | 77,08 | Paracentral_Lobule_L |
| **34** | 0,96 | 3,21 | 42,68 | Cingulum_Mid_R |
| **35** | 18,78 | -6,90 | -24,49 | ParaHippocampal_R |
| **36** | 27,84 | -90,25 | -12,50 | Lingual_R |
| **37** | 30,61 | -44,94 | -54,85 | Cerebelum_8_R |
| **38** | 32,90 | 62,56 | -8,32 | Frontal_Mid_Orb_R |
| **39** | 49,94 | -66,54 | -13,80 | Occipital_Inf_R |
| **40** | -40,50 | 9,66 | -11,41 | Insula_L |
| **41** | 0,99 | -45,98 | -20,43 | Vermis_3 |
| **42** | 39,16 | 15,44 | -13,43 | Insula_R |
| **43** | -61,70 | -16,73 | -10,29 | Temporal_Mid_L |
| **44** | -54,26 | 20,16 | 14,20 | Frontal_Inf_Tri_L |
| **45** | -48,37 | -66,32 | 31,89 | Angular_L |
| **46** | -23,23 | -19,73 | -14,12 | Hippocampus_L |
| **47** | -21,23 | 32,13 | 45,84 | Frontal_Sup_L |
| **48** | -0,39 | -55,14 | 31,66 | Precuneus_L |
| **49** | -0,30 | 58,88 | -6,32 | Frontal_Med_Orb_L |
| **50** | 5,08 | -54,03 | -48,48 | Cerebelum_9_R |
| **51** | 25,09 | -20,26 | -13,68 | Hippocampus_R |
| **52** | 22,55 | 32,33 | 49,35 | Frontal_Sup_R |
| **53** | 51,89 | -63,29 | 30,74 | Angular_R |
| **54** | 63,43 | -8,63 | -11,98 | Temporal_Mid_R |
| **55** | -66,94 | -49,28 | -1,76 | Temporal_Mid_L |
| **56** | -57,24 | -56,94 | 44,76 | Parietal_Inf_L |
| **57** | -0,79 | 32,15 | 47,25 | Frontal_Sup_Medial_L |
| **58** | 27,36 | -100,37 | 0,19 | Occipital_Mid_R |
| **59** | 58,22 | -58,31 | 40,48 | Parietal_Inf_R |
| **60** | -54,15 | -7,25 | 35,64 | Postcentral_L |
| **61** | 20,72 | -74,53 | 41,99 | Occipital_Sup_R |
| **62** | 45,25 | -19,95 | 42,58 | Postcentral_R |
| **63** | 0,60 | -50,19 | -38,85 | Vermis_9 |
| **64** | -50,99 | -29,51 | 24,20 | SupraMarginal_L |
| **65** | -31,58 | 36,25 | 28,57 | Frontal_Mid_L |
| **66** | 9,53 | -99,13 | 21,03 | Occipital_Sup_R |
| **67** | 31,25 | -74,61 | -52,93 | Cerebelum_7b_R |
| **68** | 40,08 | 31,85 | 31,10 | Frontal_Mid_R |
| **69** | 61,72 | -56,17 | -2,68 | Temporal_Inf_R |
| **70** | 0,81 | 48,14 | 15,13 | Cingulum_Ant_L |
| **71** | -61,14 | -23,80 | 24,88 | SupraMarginal_L |
| **72** | -55,76 | 7,31 | 26,56 | Precentral_L |
| **73** | -50,15 | 35,98 | 15,70 | Frontal_Inf_Tri_L |
| **74** | -19,76 | -68,37 | -55,00 | Cerebelum_8_L |
| **75** | 47,44 | 14,34 | 28,19 | Frontal_Inf_Oper_R |
| **76** | 61,13 | -26,72 | 24,17 | SupraMarginal_R |
| **77** | -44,84 | -8,73 | -39,32 | Temporal_Inf_L |
| **78** | -24,32 | -76,43 | -49,69 | Cerebelum_7b_L |
| **79** | 24,90 | -76,64 | -49,57 | Cerebelum_7b_R |
| **80** | 41,90 | -9,07 | -39,62 | Temporal_Inf_R |
| **81** | -56,46 | -45,17 | 10,36 | Temporal_Mid_L |
| **82** | 13,46 | -86,62 | 13,90 | Calcarine_R |
| **83** | 61,67 | -14,79 | 12,35 | Temporal_Sup_R |
| **84** | 7,08 | -19,81 | 27,26 | Cingulum_Mid_R |

**SUPPLEMENTARY FIGURES**

**Supplementary figure 1**: Description of the network’s components and their corresponding explained variance


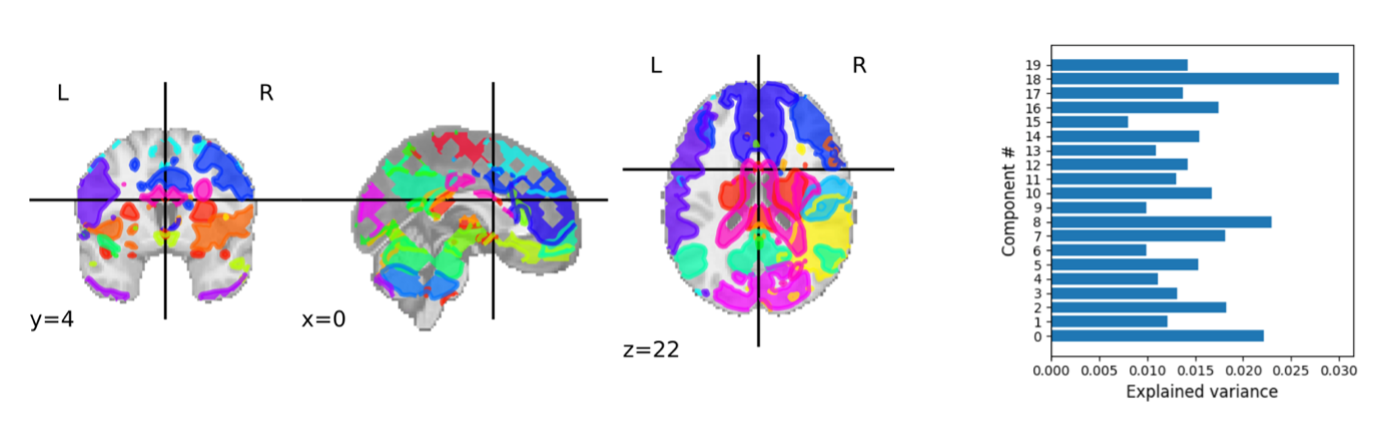


Legend: L: Left, R: Right

*Legend*: Dictionary Learning Model was fit into the rs dataset to decompose spatial maps based on sparsity. Each derived component represents a distinct functional network (see left figure) accounting for part of the explained variance in the fMRI BOLD time series (see right figure). Using Nilearn’s Regions Extractor, these networks were automatically segmented into Regions Of Interest (See supplementary Table 1) that were used to build the individual connectomes.

**Supplementary figure 2**: Illustration of the direct effect of Primary substance use at time t and Primary substance use at time t+1 and the moderating effect of Momentary Stroop Performance (group-centered).


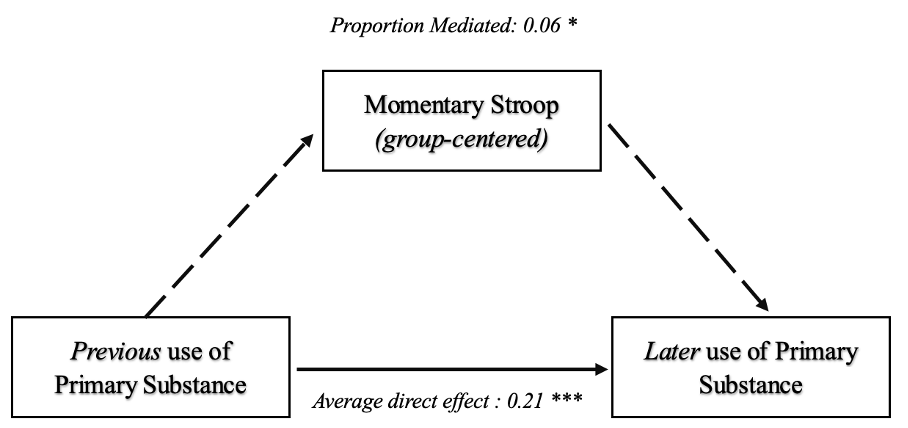


Legend: ***:p<.001, *:p<.05
